# Prostaglandin E2 Promotes Endothelial Differentiation from Bone Marrow-Derived Cells through AMPK Activation

**DOI:** 10.1371/journal.pone.0023554

**Published:** 2011-08-18

**Authors:** Zhenjiu Zhu, Chenglai Fu, Xiaoxia Li, Yimeng Song, Chenghong Li, Minghui Zou, Youfei Guan, Yi Zhu

**Affiliations:** 1 Key Laboratory of Molecular Cardiovascular Sciences of Education Ministry, Department of Physiology and Pathophysiology, Peking University Health Science Center, Beijing, China; 2 Division of Endocrinology and Diabetes, Department of Medicine, University of Oklahoma Health Sciences Center, Oklahoma City, Oklahoma, United States of America; Northwestern University, United States of America

## Abstract

Prostaglandin E2 (PGE2) has been reported to modulate angiogenesis, the process of new blood vessel formation, by promoting proliferation, migration and tube formation of endothelial cells. Endothelial progenitor cells are known as a subset of circulating bone marrow mononuclear cells that have the capacity to differentiate into endothelial cells. However, the mechanism underlying the stimulatory effects of PGE2 and its specific receptors on bone marrow-derived cells (BMCs) in angiogenesis has not been fully characterized. Treatment with PGE2 significantly increased the differentiation and migration of BMCs. Also, the markers of differentiation to endothelial cells, CD31 and von Willebrand factor, and the genes associated with migration, matrix metalloproteinases 2 and 9, were significantly upregulated. This upregulation was abolished by dominant-negative AMP-activated protein kinase (AMPK) and AMPK inhibitor but not protein kinase, a inhibitor. As a functional consequence of differentiation and migration, the tube formation of BMCs was reinforced. Along with altered BMCs functions, phosphorylation and activation of AMPK and endothelial nitric oxide synthase, the target of activated AMPK, were both increased which could be blocked by EP4 blocking peptide and simulated by the agonist of EP4 but not EP1, EP2 or EP3. The pro-angiogenic role of PGE2 could be repressed by EP4 blocking peptide and retarded in EP4^+/−^ mice. Therefore, by promoting the differentiation and migration of BMCs, PGE2 reinforced their neovascularization by binding to the receptor of EP4 in an AMPK-dependent manner. PGE2 may have clinical value in ischemic heart disease.

## Introduction

Angiogenesis is a process of new blood vessel formation from the existing vascular bed. Numerous studies have demonstrated that a population of cells mobilized from bone marrow and circulating in peripheral blood, participate in postnatal neovascularizaton [1], [2]. Both animal and clinical studies have shown that circulating bone-marrow derived cells (BMCs) are mobilized endogenously in response to tissue ischemia and thereby augment neovascularization during ischemia [3], tumor vasculature [4], wound healing [5] and inflammation [5]. These cells home to vascular injury sites, adapt to the endothelial phenotype, and contribute to angiogenesis, they was named as endothelial progenitor cells (EPCs) [1], [2]. Key determining factors of these cells are adhesion at the site of injury and differentiation into vascular endothelial cells (ECs). Although EPCs have clinical implications for therapeutic vasculogenesis, the identification of the involved cell populations and the mechanism by which they participate in vascular repair remain largely unknown. Many growth factors, cytokines and chemokines are reported to be involved in the process [3], [6]. However, there are still no specific markers for EPCs and assessment of cell purity, and results from clinical trials remain controversial [5]. Even a proteomics-led approach in early outgrowth EPCs raised the awareness that markers used to define their endothelial potential might arise from an uptake of platelet proteins [5]. Recently, Asahara's group consistently reported that mouse CD34(+) cells may represent a functional EPC population in bone marrow [5]. In this study, the term of BMCs was used to represent this group of cells.

Prostaglandin E2 (PGE2) is widely recognized as a mediator of inflammation, capable of recruiting proinflammatory cells and causing pain. PGE2 is also known to promote tumorigenesis because of its causal association with tumor growth and its ability to activate angiogenesis. Thus, PGE2 may contribute to the mobilization of BMCs and promote neovascular formation [3], [6]. PGE2 exerts its cellular effects by binding to 4 distinct transmembrane-specific G-protein–coupled receptors, namely EP 1–4. EP1 mediates PGE2-induced intracellular calcium mobilization. EP3 downregulates adenylate cyclase via Gi, thereby inhibiting cAMP formation. EP2 and EP4 couple to Gs and stimulate adenylate cyclase, thus resulting in increased intracellular cAMP formation. As well, EP4 but not EP2 couples to phosphatidylinositol 3-kinase probably via Gi. Apparently, different receptors mediate the diversified function of PGE2 in different cell types. The role of different EP receptors in the differentiation of BMCs is still elusive.

AMP-activated protein kinase (AMPK), a heteroteimeric serine/threonine protein kinase, which is activated in many cell types by increased intracellular concentrations of AMP, plays an important role in angiogenesis when activated by vascular endothelial growth factor (VEGF) [7], [8], [9], basic fibroblast growth factor [10], adiponectin [11], [12] and hypoxia [9]. We previously reported that activation of AMPK and the consequent activation of endothelial nitric oxide synthase (eNOS) play a pivotal role in the differentiation of human EPCs [13].

Because of the important role of PGE2 as a mediator in the inflammatory response and in angiogenesis, we tested the hypothesis that PGE2 may also be a stimulator of the differentiation of BMCs into mature ECs. Further, we studied the mechanism of the beneficial effects of PGE2-mediated BMC differentiation in vascular repair. Through promoting the differentiation and migration of BMCs, PGE2 reinforced their neovascularization by binding to the EP4 receptor in an AMPK-dependent pathway.

## Materials and Methods

### Reagents

PGE2; the blocking peptides for EP1, EP2, EP3, EP4; and the agonists for EP2 (Butaprost), EP3 (Sulprostone), and EP4 (PGE1 Alcohol) were from Cayman Chemical (Ann Arbor, MI). Compound C and protein kinase A (PKA) inhibitor (rAMP) were from Sigma Aldrich (St Louis, MO). EBM-2 medium was from Lonza Clonetics (Walkersville, MD), and fetal bovine serum was from Hyclone (Logan, UT). Dil-labeled acetylated low-density lipoprotein (Dil-acLDL) was from Invitrogen (Carlsbad, CA) and the Boyden chamber (8.0 µm) was from Becton Dickinson (Franklin Lakes, NJ). Anti-p-AMPK-Thr172, anti-p-eNOS-Ser-1177, anti-AMPK, and anti-eNOS antibodies were from Cell Signaling Technology (Danvers, MA). The other primary antibodies and all secondary antibodies were from Santa Cruz Biotechnology (Santa Cruz, CA). Ulex europaeus agglutinin 1 (UEA-1) and all other reagents were of tissue-culture or molecular biology grade and purchased from Sigma Aldrich.

### BMC isolation, culture, and identification

BMCs were harvested by flushing through the femoral and tibial bones and cultured with EBM-2 medium supplemented with 10% fetal bovine serum and cytokine cocktail. After culture for 7 to 10 days, clusters of BMC colonies grew at a high proliferative rate into a monolayer with a spindle-shaped morphology. This cell population appeared to be homogenous and maintained a similar morphology. The population exhibited high levels of CD34, c-kit and VEGF receptor 2 (VEGFR-2; Flk-1) and had high capacity for taking up Dil-acLDL and binding to UEA-1. The methods and results for BMC identification are in Figure S1. BMCs within passages 5 were used in all experiments.

### Cell migration and transwell assay

BMC migration was assessed by scratch-wound assay as described [14]. A scratch in a cell monolayer was created, and the resulting migration was captured at the beginning and 8 hr later. The images were quantified for the rate of cell migration. The transwell assay involved use of the Boyden chamber. In brief, 4×10^4^ cells/well was suspended in 200 µl. EBM-2 medium was loaded into the upper chamber of a transwell cluster plate. A 0.4-ml EBM-2 medium was added to the lower chamber; 1 µM PGE2 or an equal volume of DMSO was added to both chambers. After incubation for 24 hr, cells that migrated onto the lower side of the membrane were fixed with 2% paraformaldehyde and then stained with Hoechst and DiIC18, a dye for plasma membrane staining. The stained cells in each well were photographed and counted.

### In situ immunofluorescence staining

Subconfluent BMCs grown on coverslips were treated as indicated. The cells were fixed with 2% paraformaldehyde and immunostained. The primary antibodies included goat anti-CD34, rabbit anti-eNOS, rabbit anti-von Willebrand factor (vWF) or mouse anti-VE-cadherin. The secondary antibodies were FITC-conjugated anti-goat, FITC-conjugated anti-rabbit or TRITC-conjugated anti-mouse antibodies. The nuclei were counterstained with Hoechst. The results were observed under a confocal microscope.

### In vitro angiogenesis assay

In vitro angiogenesis of BMCs on Matrigel (Becton Dickinson) was as we previously described [13].

### In vivo vasculogenesis

The investigation conforms with the Guide for the Care and Use of Laboratory Animals published by the US National Institutes of Health (NIH Publication N0. 85–23, revised 1996). The animal experimental protocol was approved by the Peking University Institutional Animal Care and Use Committee (LA2011-003). Male C57BL/6 mice (8 weeks old) were fed standard laboratory chow and tap water ad libitum. The Matrigel plug *in vivo* vasculogenesis assay was as described previously [13]. The BMCs isolated from wild-type mice were pretreated with PGE2 for 24 hr, then 5×10^5^ cells were resuspended in 100 µL Matrigel on ice. The mixture was implanted on the flanks of wild-type or EP4^+/−^ mice (n = 6) by subcutaneous injection and left for 7 days [13], then implants were removed and stained with hematoxylin and eosin.

### Adenovirus infection

Ad-AMPK-DN, a recombinant adenovirus expressing a dominant-negative mutant of AMPK, was described previously [15]. Recombinant viruses were amplified, and the titers were determined in HEK293 cells. Confluent BMCs were infected with recombinant adenoviruses (50 multiplicity of infection) for 24 hr before further treatments. The parental adenoviral vector or Ad-GFP was used as an infection control [13].

### Quantitative real-time PCR and Western blot analysis

Total RNA was isolated from BMCs with use of TRIzol reagent. The resulting cDNAs were used as templates for quantitative RT-PCR with the EVA Green fluorescent DNA stain. β-actin was used as an internal control. The nucleotide sequences of the primers are in Table 1.

**Table 1:** Primers used in this study.

MOUSE	NM	Position	Product (bp)	Forward (5′ to 3′)	Reverse (5′ to 3′)
CD34	NM_001111059	+463 to +678	216	ACCACAGACTTCCCCAACTG	CGGATTCCAGAGCATTTGAT
c-kit	NM_001122733	+4580 to +4731	152	GGTATGTTGCCTTCACGGTT	CATGACAACAGGACCTCCAA
CD31	NM_008816	+1070 to +1330	261	GTCATGGCCATGGTCGAGTA	CTCCTCGGCATCTTGCTGAA
VWF	NM_011708	+8682 to +8795	114	CCTGTGCAGCTACAGCGGATTC	TTATTGTGGGCCCAGGAGGGCA
MMP2	NM_008610	+2144 to +2278	135	CTGATAACCTGGATGCCGTCGT	CCAGCCAGTCTGATTTGA
MMP9	NM_013599	+1660 to +1887	228	TTCAAGGACGGTTGGTACT	CTCTGGGCCTAGACCCAACTTA
CYP19a	NM_007810	+115 to +262	138	CACATCCTCAATACCAGGTCC	CAGAGATCCAGACTCGCATG
COX2	NM_011198	+1509 to +1702	194	AGAAGGAAATGGCTGCAGAA	GCTCGGCTTCCAGTCTTGAG
EP1	NM_013641	+331 to +976	646	TTAACCTGAGCCTAGCGGATG	CGCTGAGCGTATTGCACACTA
EP2	NM_008964	+1464 to +1589	126	ATGCTCCTGCTGCTTATCGT	AGGGCCTCTTAGGCTACTGC
EP3	NM_011196	+914 to +1131	218	GGATCATGTGTGTGCTGTCC	GCAGAACTTCCGAAGAAGGA
EP4	NM_008965	+82 to +305	224	GTTCCGAGACAGCAAAAGC	CACCCCGAAGATGAACATCAC
β-Actin	NM_007393	+809 to +948	140	GACGGCCAGGTCATCACTAT	CGGATGTCAACGTCACACTT

Western blot analysis followed standard protocols. The primary antibodies were anti-p-AMPK-Thr172, anti-p-eNOS-Ser-1177, anti-AMPK, anti-eNOS and β-actin.

### Statistical analysis

The significance of variability was determined by ANOVA with post-hoc comparison by Student's *t* test for continuous variables and by chi-square or Fisher's exact test for nominal variables, as appropriate. All results are mean ± SD from at least 3 independent experiments. P<0.05 was considered statistically significant.

## Results

### PGE2 induces the differentiation of BMCs to mature ECs

**Figure 1:**
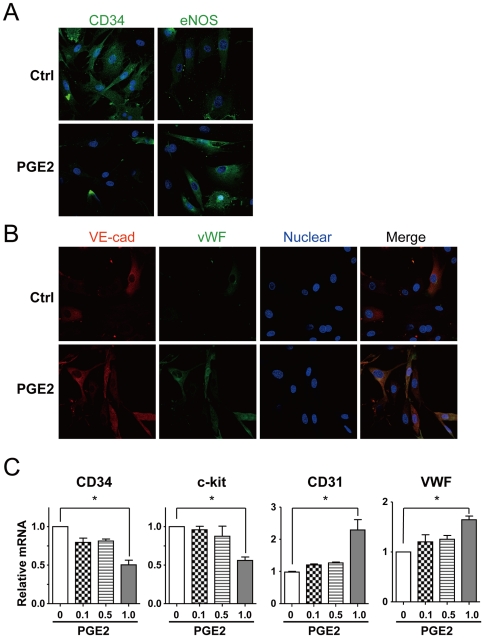
PGE2 induces the differentiation of BMCs. BMCs were pretreated with PGE2 (1 µM) for 24 hr. (A) Confocal microscopy of cells immunostained with goat-anti-CD34 or rabbit-anti-endothelial nitric oxide synthase (eNOS) primary antibodies. (B) Confocal microscopy of treated cells double-immunostained with rabbit anti-von Willebrand factor (vWF) and mouse anti-VE-cadherin primary antibodies. (C) BMCs were treated with different doses of PGE2 as indicated; the mRNA levels of CD34, c-kit, CD31, vWF were detected by quantitative RT-PCR (qRT-PCR). Data are mean ± SD of the mRNA levels normalized to that of β-actin and expressed as fold of that of control (* p<0.05).

To detect the effect of PGE2 on the differentiation of BMCs, we treated these cells with PGE2 and detected the differentiation markers of ECs by immunostaining. Figure 1A shows that CD34 was downregulated and eNOS upregulated in PGE2-treated cells. In addition, the expression of vWF and VE-cadherin, the markers of mature ECs, was increased in cells treated with PGE2 (Fig. 1B). Quantitative real-time PCR analysis revealed that the mRNA levels of the progenitor cell markers CD34 and c-kit were decreased and that of the EC markers CD31 and vWF increased in BMCs treated with PGE2 in a dose-dependent manner, especially at 1 µM (Fig. 1C).

### PGE2 promotes the abilities of migration and tube formation in BMCs

**Figure 2:**
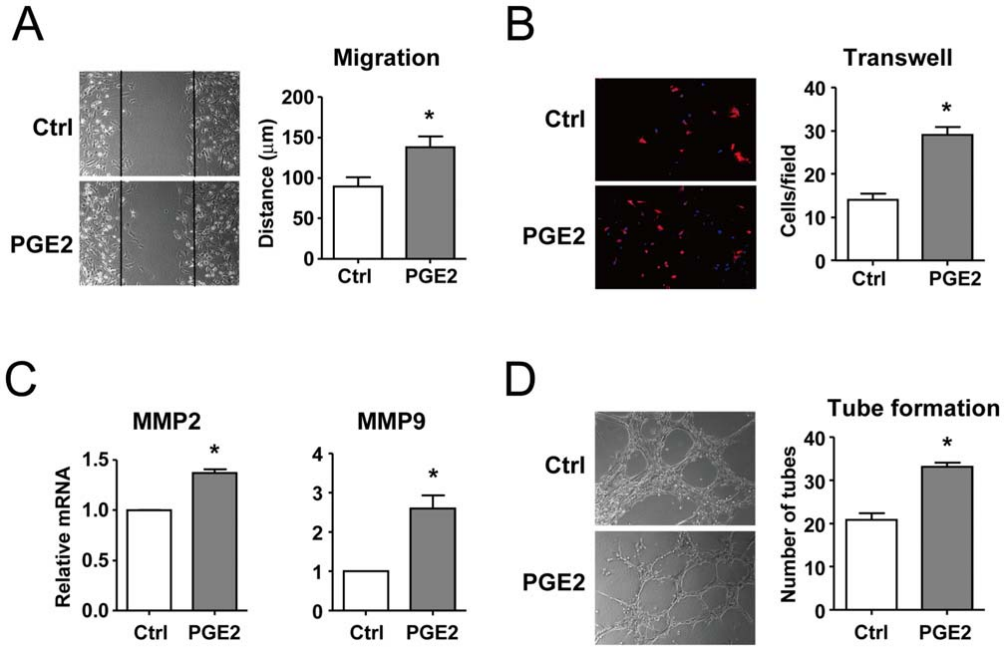
PGE2 enhanced the endothelial functions of BMCs. (A) BMCs were pretreated with PGE2 (1 µM) for 24 hr. Then, the confluent monolayers of cells were scratch wounded, and the migration distance was observed after 8 hr. The black line indicates the original wound edge. The mean distance of migration was quantified by measuring the average of 5 independent microscope fields for each of 3 independent experiments. Magnification is ×100. *P<0.05. (B) The cells migrating across the filters were stained. Magnification is ×100. Representative results of 3 independent experiments are shown. Migrated cells were quantified by the average of 4 randomly chosen high-power fields of 3 independent experiments, each performed in duplicate. Data are mean±SD, *P<0.05. (C) BMCs were treated with PGE2 (1 µM) for 24 hr. mRNA levels of MMP2 and MMP9 were examined by qRT-PCR. Data are mean ± SD of the mRNA levels normalized to that of β-actin and expressed as fold of control (* p<0.05). (D) BMCs were pretreated with PGE2 (1 µM) for 24 hr. Then, 1×10^5^ cells were plated onto Matrigel for 4 hr. Tube formation was determined by counting the number of tubes in 5 randomly chosen low-power fields. Data are mean±SD from 3 independent experiments, each performed in triplicate (*P<0.05).

BMCs can be recruited to sites of neovascularization where they differentiate into mature ECs *in situ*. We further investigated whether PGE2 can induce BMC migration and increase tube formation. Results from both scratch-wound and trans-well assays showed that PGE2 increased the migration of BMCs (Fig. 2A,B). This effect of PGE2 was not due to increased cell proliferation because PGE2 did not change the cell proliferation index or cell cycle (Fig.S2). We then detected the expression of matrix metalloproteinases 2 and 9 (MMP2 and MMP9), which is associated with migration ability. Real-time PCR results showed that PGE2 upregulated the expression of MMP2 and MMP9 (Fig. 2C). Further, *in vitro* tube-formation assay revealed that PGE2 exposure significantly increased the tube formation of BMCs on Matrigel (Fig. 2D).

### The differentiation effects of PGE2 on BMCs depended on AMPK activation

**Figure 3:**
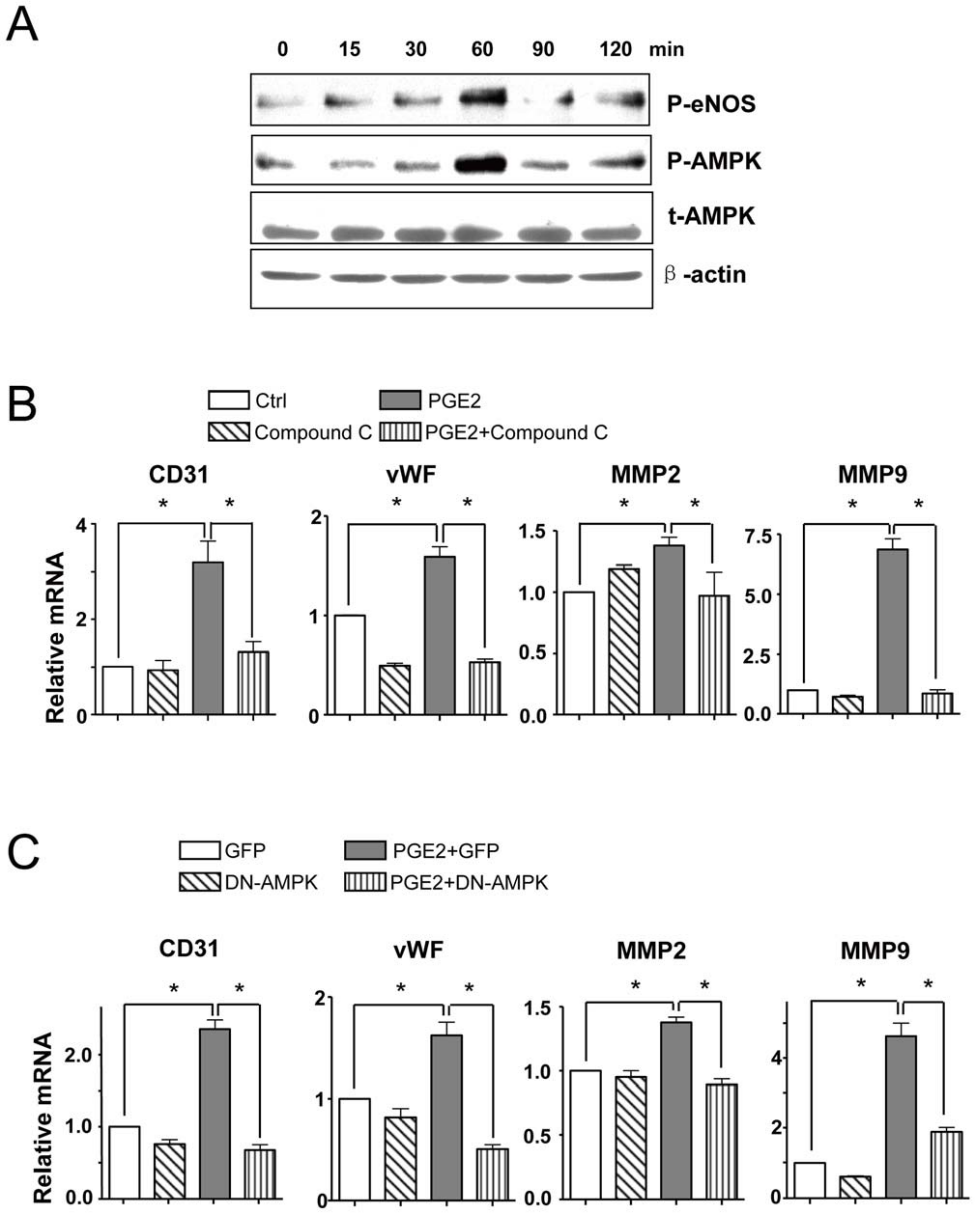
PGE2 mediated the differentiation and migration of BMCs through AMPK activation. (A) BMCs were treated with PGE2 (1 µM) for different times as indicated, and protein extracts were pooled and examined by western blot analysis. (B) BMCs were pretreated with Compound C (10 µM) for 30 min, then cells were treated with PGE2 for 24 hr. (C) BMCs was pretreated without or with an adenovirus expressing a dominant-negative mutant of AMPK (Ad-AMPK-DN, 50 multiplicities of infection) for 24 hr, then infected cells were treated with PGE2 for another 24 hr. (B, C) Total mRNA was isolated and analyzed by qRT-PCR.

**Figure 4:**
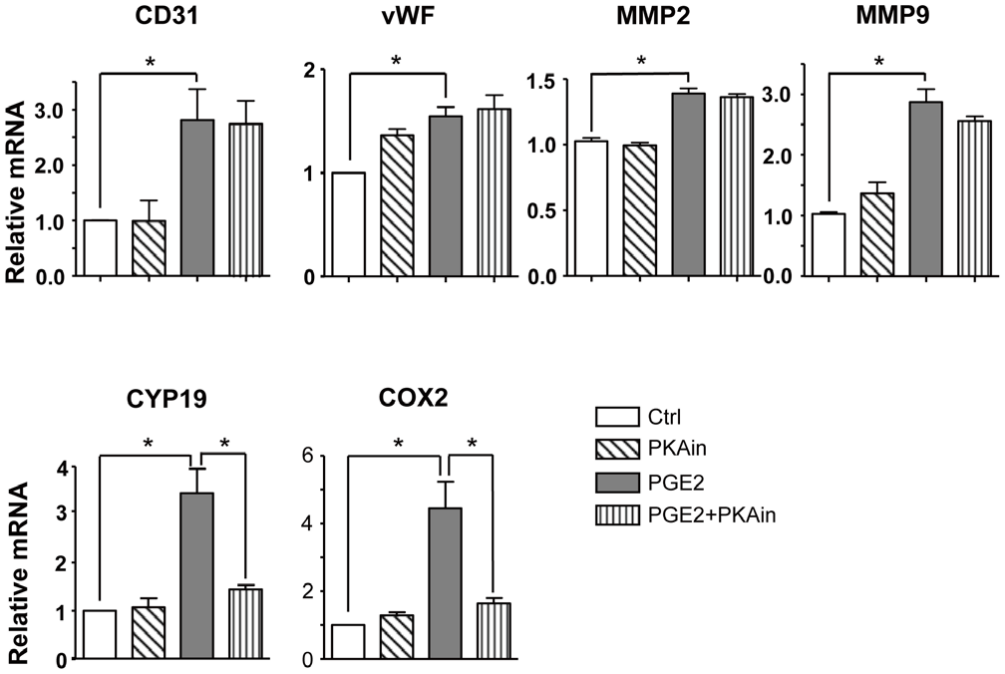
Protein kinase A (PKA) has no effect on differentiation and migration of BMCs mediated by PGE2. BMCs was pretreated with PKA inhibitor rAMP (PKAin; 10 µM) for 30 min, then cells were treated with PGE2 (1 µM) for 24 hr. Total mRNA was isolated and analyzed by real-time RT-PCR for expression of CD31, vWF, MMP2, MMP9, CYP19 and COX2.

We previously reported that AMPK plays a pivotal role in the differentiation of BMCs [13]. We tested whether AMPK also has a role in the differentiation of BMCs induced by PGE2. On treatment with PGE2 from 15 min to 2 hr, the phosphorylation of AMPKα at Thr172 peaked at 60 min; as well, the phosphorylation of eNOS at Ser1177, a target of activated AMPK, peaked at 60 min (Fig. 3A). The activation of AMPK blocked with Compound C, a chemical activity inhibitor of AMPK, and DN-AMPK, suppressed the increased mRNA levels of CD31, vWF, MMP2 and MMP9 by PGE2 (Fig. 3B,C). As previously described, PGE2 was able to activate PKA [16], [17], [18], but we found that rAMP, a PKA inhibitor, did not alter the effect of PGE2 on the differentiation and migration of BMCs (Fig. 4A) but did decrease the expression of the positive controls CYP19 [19] and COX-2 [16], [17], [18], both reported to be upregulated by PGE2 through the PKA-CREB pathway (Fig. 4B).

### PGE2 effect was through its receptor EP4

**Figure 5:**
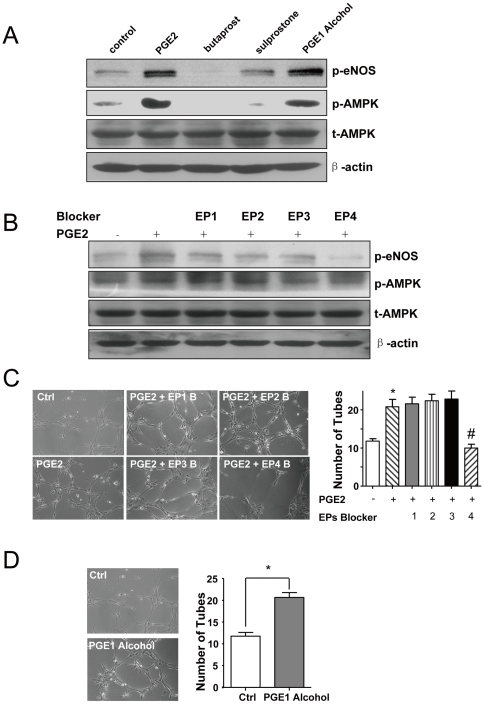
PGE2 mediated vasculogenesis of BMCs via EP4 receptor. (A) BMCs was pretreated with or without butaprost (1 µM), sulprostone (1 µM), PGE1 alcohol (1 µM) and (B) blocking peptides of EP1, EP2, EP3 and EP4 in for 15 min, then cells were treated with PGE2 (1 µM) for 60 min. The total protein extracts were pooled, and the phosphorylation of AMPK and eNOS was examined by western blot analysis. BMCs were treated without or with the blocking peptides and then treated with PGE2 in (C) and PGE1 alcohol in (D) for 24 hr for tube-formation assay as described in Figure 2D. Data are means ± SD from 3 independent experiments, each performed in triplicate (*P<0.05).

**Figure 6:**
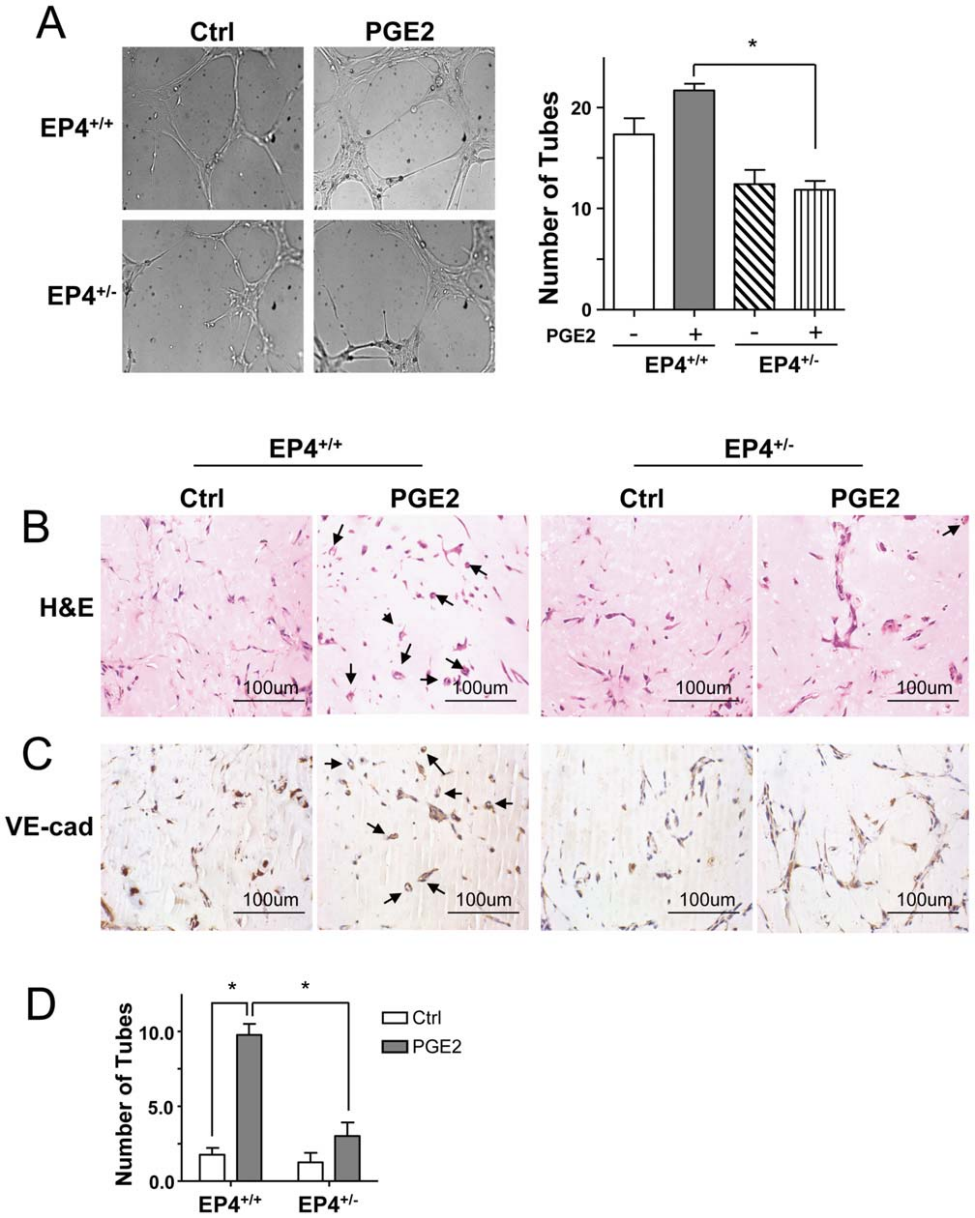
The vasculogenesis of BMCs mediated by PGE2 is attenuated in EP4^+/−^ mice. BMCs from EP4^+/−^ mice or their wild-type littermates were treated with PGE2 (1 µM) for 24 hr. (A) tube-formation assay was performed as described in Figure 2D. (B) and (C) cells mixed with Matrigel were subcutaneously injected into C57BL/6 mice and kept for 7 days. (B) The cross sections of the implants underwent hematoxylin and eosin (H&E) staining, and (C) immunohistochemical staining with anti-VE-cadherin (VE-Cad). Magnification ×400. The arrows indicate vessel-like structures. All images are representative of implants from 6 different animals. (D) Microvessel density in Matrigel implants quantified by counting luminal structures containing erythrocytes. Data are mean±SD microvessel density value determined from 3 different implants in 2 independent experiments *P<0.05.

PGE2 exerts its cellular effects through binding to its 4 distinct transmembrane-specific G-protein-coupled receptors. We investigated which receptor mediated the effect of PGE2 in BMC differentiation. We first detected the expression level of different EPs in cultured BMCs and found that the expression of EP1 and EP4 was higher than that of EP2 and 3, with the expression of EP4 the highest (Fig. S3). Then, we studied the role of different EPs in mediating the activation of AMPK and tube formation. As shown in Figure 5A, PGE1 alcohol, an EP4 agonist, but not the EP2 agonist butaprost or EP3/EP1 agonist sulprostone induced the phosphorylation of AMPK and eNOS. Further, we found that the phosphorylation of AMPK and eNOS induced by PGE2 was inhibited by EP4 blocking peptide but not the other 3 blocking peptides (Fig. 5B). The EP4 blocking peptide but not EP1–3 blocking peptides repressed the pro-angiogenic tube-formation effect of PGE2 in BMCs (Fig. 5C). However, the EP4 agonist could mimic the effect of PGE2 alcohol and simulate tube formation (Fig. 5D). To further confirm the role of EP4 mediating the effect of PGE2 in tube formation, BMCs were isolated from EP4^+/−^ mice and their wild-type littermates, EP4^+/+^ mice. Tube formation was lower in EP4^+/−^ cells than in EP4^+/+^ cells. The response of PGE2 in EP4^+/−^ cells was also largely diminished (Fig. 6A).

### PGE2 enhanced the vasculogenesis of BMCs in vivo

To relate our *in vitro* results with BMC function and angiogensis *in vivo*, we observed capillary formation in Matrigel plugs subcutaneously implanted in mice. BMCs from EP4^+/−^ and EP4^+/+^ mice were pretreated with PGE2 and then resuspended with Matrigel and injected subcutaneously into C57BL/6 mice. After 7 days, Matrigel implants were removed, and hematoxylin and eosin staining revealed more luminal structure in implants with PGE2-treated EP4^+/+^ BMCs than in untreated control or PGE2-pretreated EP4^+/−^ BMCs (Fig. 6B). To further characterize the differentiation of these BMCs, VE-cadherin staining of implants showed cells in the luminal structure positive for VE-cadherin, for mature ECs (Fig. 6C). The number of luminal structures was significantly higher in PGE2-treated EP4^+/+^ cells than in PGE2-treated EP4^+/−^ cells (Fig. 6D). Furthermore, the increasing luminal structures by PGE2 mainly from the injected BMCs rather than cells from the host mice (Fig. S4). Therefore, PGE2 induced the differentiation of BMCs *via* the EP4 receptor.

## Discussion

PGE2 has been reported to modulate angiogenesis, the process of new blood vessel formation, by promoting BMC proliferation, migration and tube formation. We investigated the mechanism underlying the stimulatory effects of PGE2 and its specific receptors on angiogenesis and BMCs. We found that 1) PGE2 induced the differentiation and migration of BMCs, which was followed by the upregulation of CD31 and vWF, markers of differentiation to ECs, and the genes associated with migration, MMP2 and MMP9; 2) the effects of differentiation of BMCs were mediated by the activation of AMPK; and 3) the activation of AMPK, the differentiation and migration, and the ability for tube formation of BMCs induced by PGE2 were through its receptor EP4.

EPCs, a minor subpopulation of peripheral blood mononuclear cells first discovered by Asahara [1], [2], [20], are believed to be derived from bone marrow progenitor cells, hematopoietic stem cells and tissue resident stem cells [21]. A proteomic analysis revealed that this population of cells might be contaminated by platelet microparticles in cultures, which acquired “endothelial” characteristics (CD31, von Willebrand factor, lectin-binding), and angiogenic properties [22]. CD34, CD31 [23] and FLK1 [24], [25] are still widely accepted as the identification markers of EPCs. The BMCs used in our study were isolated from mice bone marrow and had high capacity for uptake of Dil-acLDL and binding to UEA-1, demonstrated by immunofluorescence staining. Flow cytometry assay exhibited high levels of UEA (95.6%), FLK1 (63.04%), c-kit (47.67%) and CD31 (41.25%) in our isolated and cultured BMCs. Many studies demonstrated that EPCs participate in such repair processes as myocardial ischemia/infarction [26], endothelial repair [27], limb ischemia [28], and wound healing [29]. Circulating EPCs engraft into 15% to 29% of vessels of the transplanted human heart [30], [31]. EPCs contribute significantly to angiogenic growth-factor–induced neovascularization and may account for 26% of all ECs [32]. The population of cells in this study should have similar characteristics of above mentioned EPCs. During differentiation, BMCs gradually lose their original hematopoietic markers such as c-Kit, CD34 and CD133 and begin to express markers of mature ECs such as CD31, eNOS, and vWF. However, markers for EPCs lack clear definition. In line with the above evidence, we chose c-Kit and CD34 as markers of BMCs, and CD31 and vWF as markers of mature ECs. In addition, we analyzed BMCs by FACS with UEA, FLK1 and c-Kit for purity (Fig. S1). The mobilization and migration of BMCs, regulated by many factors, can be a complicated process. Upregulating and activating MMPs promote the mobilization of BMCs from bone marrow [33], [34] and their migration to the ischemia region [35]. Indeed, we found upregulated MMP2 and MMP9 accompanied by the migration of BMCs.

PGE2, a metabolite of arachidonic acid, was originally discovered along with other prostanoids to act on blood vessels. It is widely recognized as a mediator of inflammation, capable of recruiting proinflammatory cells and causing pain. PGE2 has also been considered a promoter of tumorigenesis because of its causal association with tumor growth [36], [37], [38]. This action of PGE2 has been attributed to its ability to activate the angiogenic switch, a process leading to angiogenesis [39]. PGE2 has been reported to promote angiogenesis, probably by increasing proliferation, migration and tube formation of BMCs. However, the mechanism of the action is not yet fully elucidated. We previously reported that activation of AMPK by VEGF and statins promoted the differentiation of human cord-blood–derived EPCs into mature ECs via an NO-dependent mechanism [13]. Here, we show that PGE2 increased the phosphorylation of AMPK and its downstream molecule eNOS. Blocking the activation of AMPK with DN-AMPK and Compound C attenuated the effect of PGE2 promoting BMC differentiation into mature ECs (Fig. 3). This finding is consistent with a previous report that the activation of AMPK by PGE2 mediated COX2 expression in renal podocytes [40]. However, PGE2 was also reported to negatively regulate AMPK by a PKA-dependent mechanism in osteoblastic MG63 cells cultured in serum-deprived media [41], which suggests different results for different conditions in different cell types. PGE2 is known to activate the PKA-CREB pathway in many cells [16], [17], [18]. In our study, PGE2 indeed caused PKA activation, as evidenced by upregulation of CYP19 [19] and COX-2 [16], [17], [18], both reported to be upregulated by PGE2 through PKA-CREB pathway. rAMP, a PKA inhibitor, blocked the induction of COX-2 and CYP19 by PGE2 but had little inhibitory effect on the upregulation of genes related to differentiation and migration, namely, CD31, vWF, MMP2 and MMP9 (Fig. 4). Hence, PGE2 may activate both PKA and AMPK pathways, but the action of promoting the differentiation of BMCs is through activating AMPK.

PGE2 has been shown to exert its cellular effects by binding to 4 distinct transmembrane receptors, namely, EP1–4. EP4 receptor plays a critical role in PGE2-dependent *in vitro* migration of ECs, and EP4 agonists induce increased vascularization *in vivo* [6]. EP4 also mediates the effect of PGE2 on angiogenesis in fracture healing [42], ocular angiogenesis [38] and retinopathy. However, Finetti et al. reported that PGE2 synergized with fibroblast growth factor 2 to promote angiogenesis mainly through reinforcing proliferation via the EP3 receptor in ECs [39]. EP3 was also found to mediate the effect of PGE2 in tumors [43], chronic inflammation [5], and wound healing [29]. EP2 was reported to mediate pulmonary angiogenesis in a murine model of emphysema [44]. Our results showed that the EP4 blocking peptide inhibited the pro-angiogenic effect of PGE2 and the EP4 agonist could mimic the effect of PGE2. Importantly, the basic and PGE2-promoted ability of tube formation of BMCs from EP4^+/−^ cells was lower than in cells from their wild-type littermates (Fig. 6). Because the ductus arteriosus fails to close after birth in EP4^−/−^ mice, thus resulting in neonatal death [45], [46], we used the heterozygous mice in this study. In line with the *in vitro* study, BMCs from EP4^+/−^ mice showed less tube formation than their wild-type littermates *in vivo*. Thus, through promoting the differentiation of BMCs, PGE2 reinforced the process of angiogenesis by binding to its receptor EP4 in an AMPK-dependent mechanism. This finding indicates that EP4 may be a potential target for promoting the differentiation of BMCs to mature ECs for clinical therapy in ischemic heart diseases.

In conclusion, PGE2 reinforced their neovascularization by promoting the differentiation and migration of BMCs. This effect was mediated by binding to the receptor of EP4 in an AMPK-dependent manner. Thus, PGE2 upregulation may have clinical value in ischemic heart disease.

## Supporting Information

Figure S1 **Identification of bone-marrow–derived cells (BMCs).** After 2 passages, (A) Confocal microscopy of BMCs were treated with Dil-labeled acetylated low-density lipoprotein (Dil-acLDL; red) for 4 hr at 37°C before binding of Ulex europeus agglutinin (UEA; green) for 1 hr and stained with Hoechst (blue). (B) BMCs were stained with UEA and antibodies against FITC-conjugated anti-mouse c-kit, FITC-conjugated anti-mouse FLK1, FITC-conjugated and FITC-labeled UEA and analyzed by FACS. Data are representative of 3 separate experiments.
(TIF)

Figure S2 **Prostoglandin E2 (PGE2) has no effect on the proliferation of BMCs.** BMCs were treated with PGE2 for 24 hr. (A) Cell cycle assay and (B) sulforhodamine B (SRB) assay was performed as indicated. The results are representative of 3 separate experiments.
(TIF)

Figure S3 **The relative expression level of different EPs in BMCs.** The BMCs were harvested by extracting total RNA for quantitative RT-PCR with primers of different EPs.
(TIF)

Figure S4 **PGE2 enhanced the vasculogenesis of BMCs in vivo.** After infection with GFP adenovirus, BMCs from C57BL/6 mice were administered with PGE2 for 24 hr. Cells mixed with Matrigel were subcutaneously injected into C57BL/6 mice and kept for 7 days, then implants were removed and GFP positive cells in frozen sections were observed under fluorescence microscopy.
(TIF)

## References

[1] Asahara T, Murohara T, Sullivan A, Silver M, van der Zee R (1997). Isolation of putative progenitor endothelial cells for angiogenesis.. Science.

[2] Ribatti D (2007). The discovery of endothelial progenitor cells. An historical review.. Leuk Res.

[3] Takahashi T, Kalka C, Masuda H, Chen D, Silver M (1999). Ischemia- and cytokine-induced mobilization of bone marrow-derived endothelial progenitor cells for neovascularization.. Nat Med.

[4] Peters BA, Diaz LA, Polyak K, Meszler L, Romans K (2005). Contribution of bone marrow-derived endothelial cells to human tumor vasculature.. Nat Med.

[5] Ueno T, Suzuki T, Oikawa A, Hosono K, Kosaka Y (2010). Recruited bone marrow cells expressing the EP3 prostaglandin E receptor subtype enhance angiogenesis during chronic inflammation.. Biomed Pharmacother.

[6] Rao R, Redha R, Macias-Perez I, Su Y, Hao C (2007). Prostaglandin E2-EP4 receptor promotes endothelial cell migration via ERK activation and angiogenesis in vivo.. J Biol Chem.

[7] Reihill JA, Ewart MA, Hardie DG, Salt IP (2007). AMP-activated protein kinase mediates VEGF-stimulated endothelial NO production.. Biochem Biophys Res Commun.

[8] Stahmann N, Woods A, Spengler K, Heslegrave A, Bauer R (2010). Activation of AMP-activated protein kinase by vascular endothelial growth factor mediates endothelial angiogenesis independently of nitric-oxide synthase.. J Biol Chem.

[9] Nagata D, Mogi M, Walsh K (2003). AMP-activated protein kinase (AMPK) signaling in endothelial cells is essential for angiogenesis in response to hypoxic stress.. J Biol Chem.

[10] Webler AC, Michaelis UR, Popp R, Barbosa-Sicard E, Murugan A (2008). Epoxyeicosatrienoic acids are part of the VEGF-activated signaling cascade leading to angiogenesis.. Am J Physiol Cell Physiol.

[11] Shibata R, Ouchi N, Kihara S, Sato K, Funahashi T (2004). Adiponectin stimulates angiogenesis in response to tissue ischemia through stimulation of amp-activated protein kinase signaling.. J Biol Chem.

[12] Ouchi N, Kobayashi H, Kihara S, Kumada M, Sato K (2004). Adiponectin stimulates angiogenesis by promoting cross-talk between AMP-activated protein kinase and Akt signaling in endothelial cells.. J Biol Chem.

[13] Li X, Han Y, Pang W, Li C, Xie X (2008). AMP-activated protein kinase promotes the differentiation of endothelial progenitor cells.. Arterioscler Thromb Vasc Biol.

[14] Wang L, Zheng J, Bai X, Liu B, Liu CJ (2009). ADAMTS-7 mediates vascular smooth muscle cell migration and neointima formation in balloon-injured rat arteries.. Circ Res.

[15] Zhang Y, Lee TS, Kolb EM, Sun K, Lu X (2006). AMP-activated protein kinase is involved in endothelial NO synthase activation in response to shear stress.. Arterioscler Thromb Vasc Biol.

[16] Pino MS, Nawrocki ST, Cognetti F, Abruzzese JL, Xiong HQ (2005). Prostaglandin E2 drives cyclooxygenase-2 expression via cyclic AMP response element activation in human pancreatic cancer cells.. Cancer Biol Ther.

[17] Ansari KM, Sung YM, He G, Fischer SM (2007). Prostaglandin receptor EP2 is responsible for cyclooxygenase-2 induction by prostaglandin E2 in mouse skin.. Carcinogenesis.

[18] Steinert D, Kuper C, Bartels H, Beck FX, Neuhofer W (2009). PGE2 potentiates tonicity-induced COX-2 expression in renal medullary cells in a positive feedback loop involving EP2-cAMP-PKA signaling.. Am J Physiol Cell Physiol.

[19] Subbaramaiah K, Hudis C, Chang SH, Hla T, Dannenberg AJ (2008). EP2 and EP4 receptors regulate aromatase expression in human adipocytes and breast cancer cells. Evidence of a BRCA1 and p300 exchange.. J Biol Chem.

[20] Li Calzi S, Neu MB, Shaw LC, Kielczewski JL, Moldovan NI (2010). EPCs and pathological angiogenesis: when good cells go bad.. Microvasc Res.

[21] Kirton JP, Xu Q (2010). Endothelial precursors in vascular repair.. Microvascular research.

[22] Prokopi M, Pula G, Mayr U, Devue C, Gallagher J (2009). Proteomic analysis reveals presence of platelet microparticles in endothelial progenitor cell cultures.. Blood.

[23] Kim H, Cho HJ, Kim SW, Liu B, Choi YJ (2010). CD31+ cells represent highly angiogenic and vasculogenic cells in bone marrow: novel role of nonendothelial CD31+ cells in neovascularization and their therapeutic effects on ischemic vascular disease.. Circulation research.

[24] Yang J, Ii M, Kamei N, Alev C, Kwon SM (2011). CD34 Cells Represent Highly Functional Endothelial Progenitor Cells in Murine Bone Marrow.. PloS one.

[25] Marrotte EJ, Chen DD, Hakim JS, Chen AF (2010). Manganese superoxide dismutase expression in endothelial progenitor cells accelerates wound healing in diabetic mice.. The Journal of clinical investigation.

[26] Hamada H, Kim MK, Iwakura A, Ii M, Thorne T (2006). Estrogen receptors alpha and beta mediate contribution of bone marrow-derived endothelial progenitor cells to functional recovery after myocardial infarction.. Circulation.

[27] Ii M, Takeshita K, Ibusuki K, Luedemann C, Wecker A (2010). Notch signaling regulates endothelial progenitor cell activity during recovery from arterial injury in hypercholesterolemic mice.. Circulation.

[28] Kwon SM, Eguchi M, Wada M, Iwami Y, Hozumi K (2008). Specific Jagged-1 signal from bone marrow microenvironment is required for endothelial progenitor cell development for neovascularization.. Circulation.

[29] Kamoshita E, Ikeda Y, Fujita M, Amano H, Oikawa A (2006). Recruitment of a prostaglandin E receptor subtype, EP3-expressing bone marrow cells is crucial in wound-induced angiogenesis.. Am J Pathol.

[30] Minami E, Laflamme MA, Saffitz JE, Murry CE (2005). Extracardiac progenitor cells repopulate most major cell types in the transplanted human heart.. Circulation.

[31] Kaye DM, Esler MD (2006). Letter by Kaye and Esler regarding article “extracardiac progenitor cells repopulate most major cell types in the transplanted human heart”.. Circulation.

[32] Murayama T, Tepper OM, Silver M, Ma H, Losordo DW (2002). Determination of bone marrow-derived endothelial progenitor cell significance in angiogenic growth factor-induced neovascularization in vivo.. Exp Hematol.

[33] Jujo K, Hamada H, Iwakura A, Thorne T, Sekiguchi H (2010). CXCR4 blockade augments bone marrow progenitor cell recruitment to the neovasculature and reduces mortality after myocardial infarction.. Proc Natl Acad Sci U S A.

[34] Jin F, Zhai Q, Qiu L, Meng H, Zou D (2008). Degradation of BM SDF-1 by MMP-9: the role in G-CSF-induced hematopoietic stem/progenitor cell mobilization.. Bone Marrow Transplant.

[35] Cui X, Chen J, Zacharek A, Li Y, Roberts C (2007). Nitric oxide donor upregulation of stromal cell-derived factor-1/chemokine (CXC motif) receptor 4 enhances bone marrow stromal cell migration into ischemic brain after stroke.. Stem Cells.

[36] Uefuji K, Ichikura T, Mochizuki H (2000). Cyclooxygenase-2 expression is related to prostaglandin biosynthesis and angiogenesis in human gastric cancer.. Clin Cancer Res.

[37] Fukuda R, Kelly B, Semenza GL (2003). Vascular endothelial growth factor gene expression in colon cancer cells exposed to prostaglandin E2 is mediated by hypoxia-inducible factor 1.. Cancer Res.

[38] Yanni SE, Barnett JM, Clark ML, Penn JS (2009). The role of PGE2 receptor EP4 in pathologic ocular angiogenesis.. Invest Ophthalmol Vis Sci.

[39] Finetti F, Donnini S, Giachetti A, Morbidelli L, Ziche M (2009). Prostaglandin E(2) primes the angiogenic switch via a synergic interaction with the fibroblast growth factor-2 pathway.. Circ Res.

[40] Faour WH, Gomi K, Kennedy CR (2008). PGE(2) induces COX-2 expression in podocytes via the EP(4) receptor through a PKA-independent mechanism.. Cell Signal.

[41] Funahashi K, Cao X, Yamauchi M, Kozaki Y, Ishiguro N (2009). Prostaglandin E2 negatively regulates AMP-activated protein kinase via protein kinase A signaling pathway.. Prostaglandins Other Lipid Mediat.

[42] Xie C, Liang B, Xue M, Lin AS, Loiselle A (2009). Rescue of impaired fracture healing in COX-2−/− mice via activation of prostaglandin E2 receptor subtype 4.. Am J Pathol.

[43] Ogawa Y, Suzuki T, Oikawa A, Hosono K, Kubo H (2009). Bone marrow-derived EP3-expressing stromal cells enhance tumor-associated angiogenesis and tumor growth.. Biochem Biophys Res Commun.

[44] Tsuji T, Aoshiba K, Yokohori N, Nagai A (2009). A systemically administered EP2 receptor agonist stimulates pulmonary angiogenesis in a murine model of emphysema.. Prostaglandins Other Lipid Mediat.

[45] Segi E, Sugimoto Y, Yamasaki A, Aze Y, Oida H (1998). Patent ductus arteriosus and neonatal death in prostaglandin receptor EP4-deficient mice.. Biochem Biophys Res Commun.

[46] Nguyen M, Camenisch T, Snouwaert JN, Hicks E, Coffman TM (1997). The prostaglandin receptor EP4 triggers remodelling of the cardiovascular system at birth.. Nature.

