# Supplementary figures and images for: Prostaglandin E2 Promotes Endothelial Differentiation from Bone Marrow-Derived Cells through AMPK Activation
Source: PLoS One. 2011 Aug 18;6(8):e23554. doi: 10.1371/journal.pone.0023554 (PMC3158081; doi:10.1371/journal.pone.0023554)

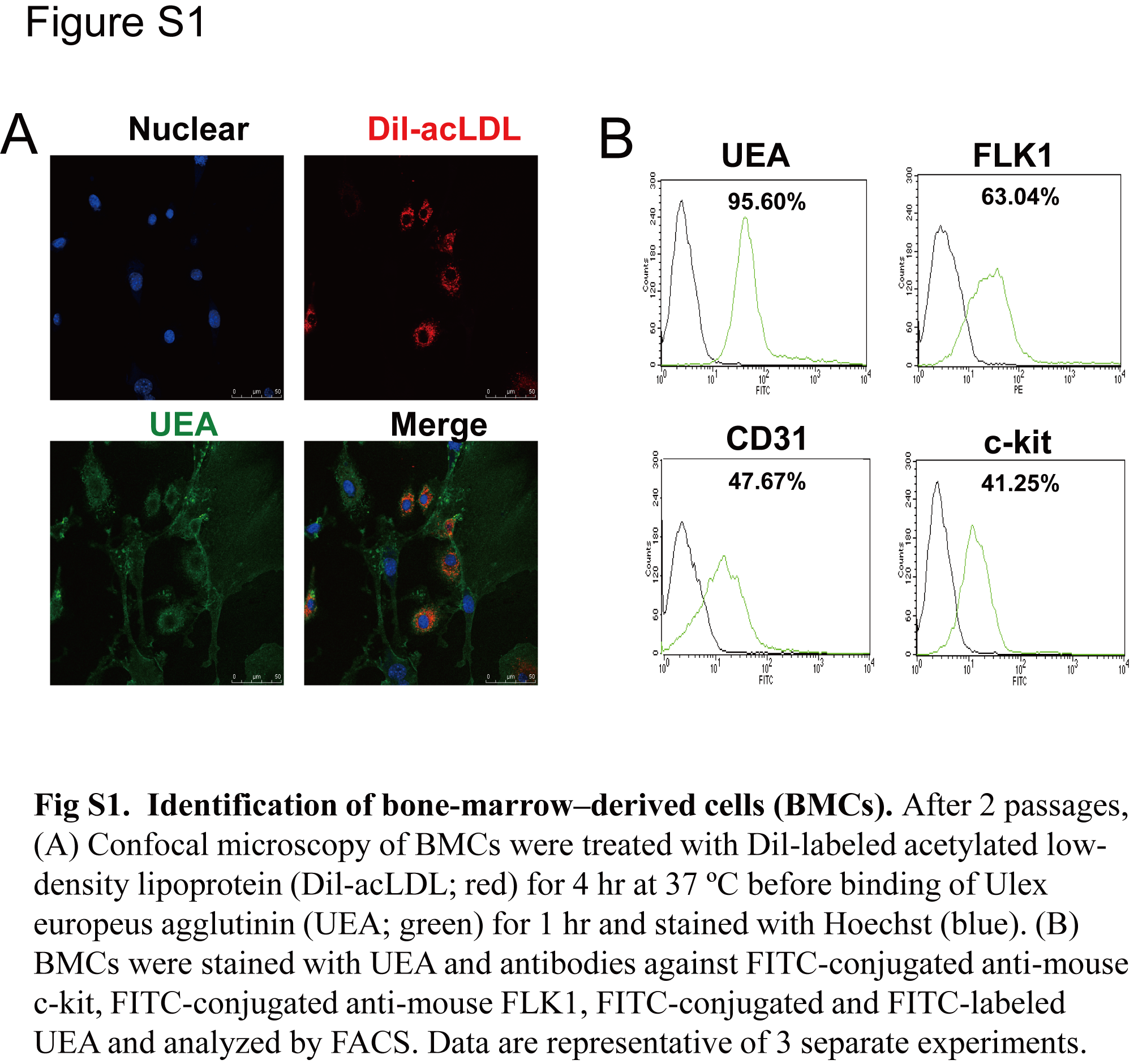

Supplement: Figure S1 — Identification of bone-marrow–derived cells (BMCs). After 2 passages, (A) Confocal microscopy of BMCs were treated with Dil-labeled acetylated low-density lipoprotein (Dil-acLDL; red) for 4 hr at 37°C before binding of Ulex europeus agglutinin (UEA; green) for 1 hr and stained with Hoechst (blue). (B) BMCs were stained with UEA and antibodies against FITC-conjugated anti-mouse c-kit, FITC-conjugated anti-mouse FLK1, FITC-conjugated and FITC-labeled UEA and analyzed by FACS. Data are representative of 3 separate experiments. (TIF) [file pone.0023554.s001.tif]

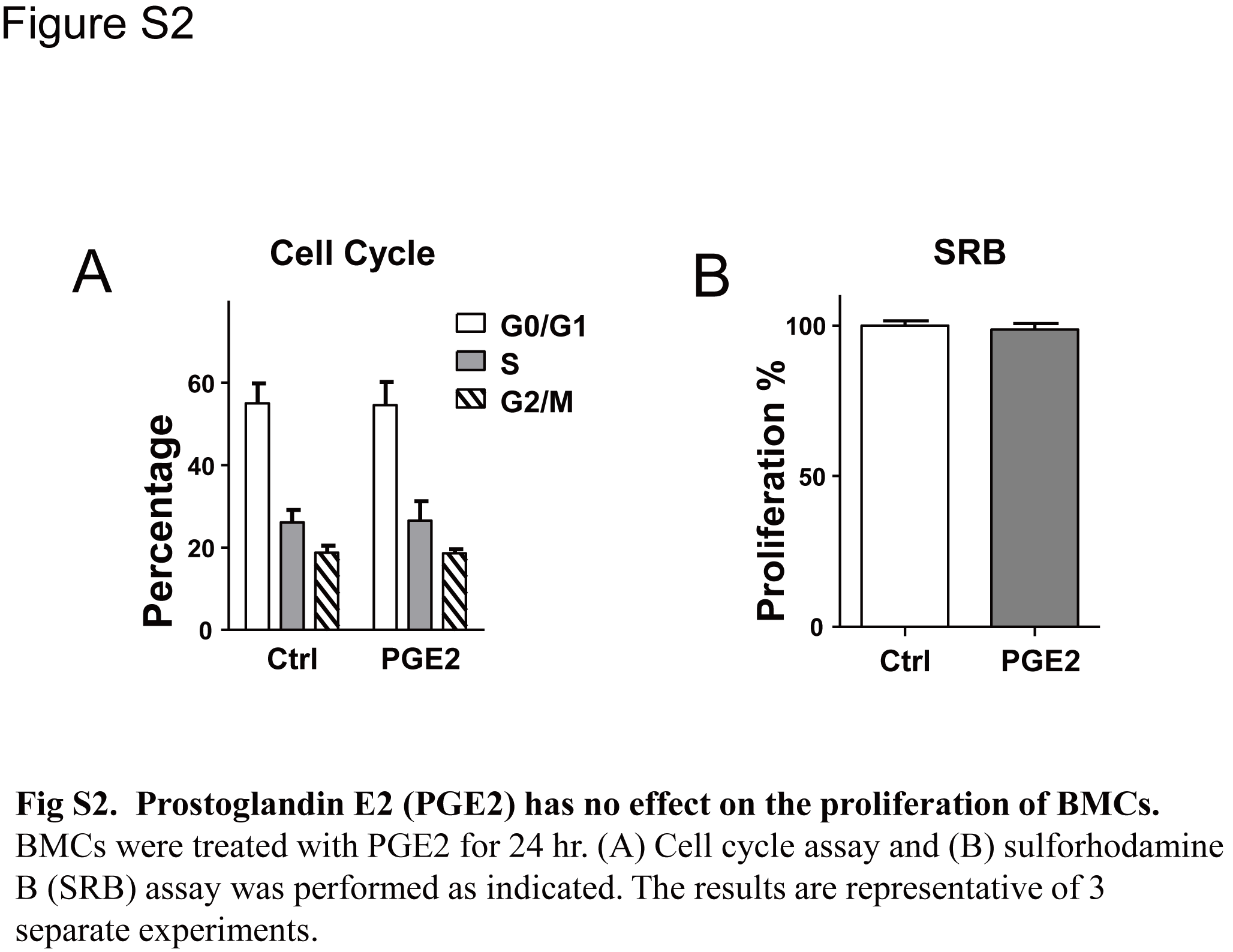

Supplement: Figure S2 — Prostoglandin E2 (PGE2) has no effect on the proliferation of BMCs. BMCs were treated with PGE2 for 24 hr. (A) Cell cycle assay and (B) sulforhodamine B (SRB) assay was performed as indicated. The results are representative of 3 separate experiments. (TIF) [file pone.0023554.s002.tif]

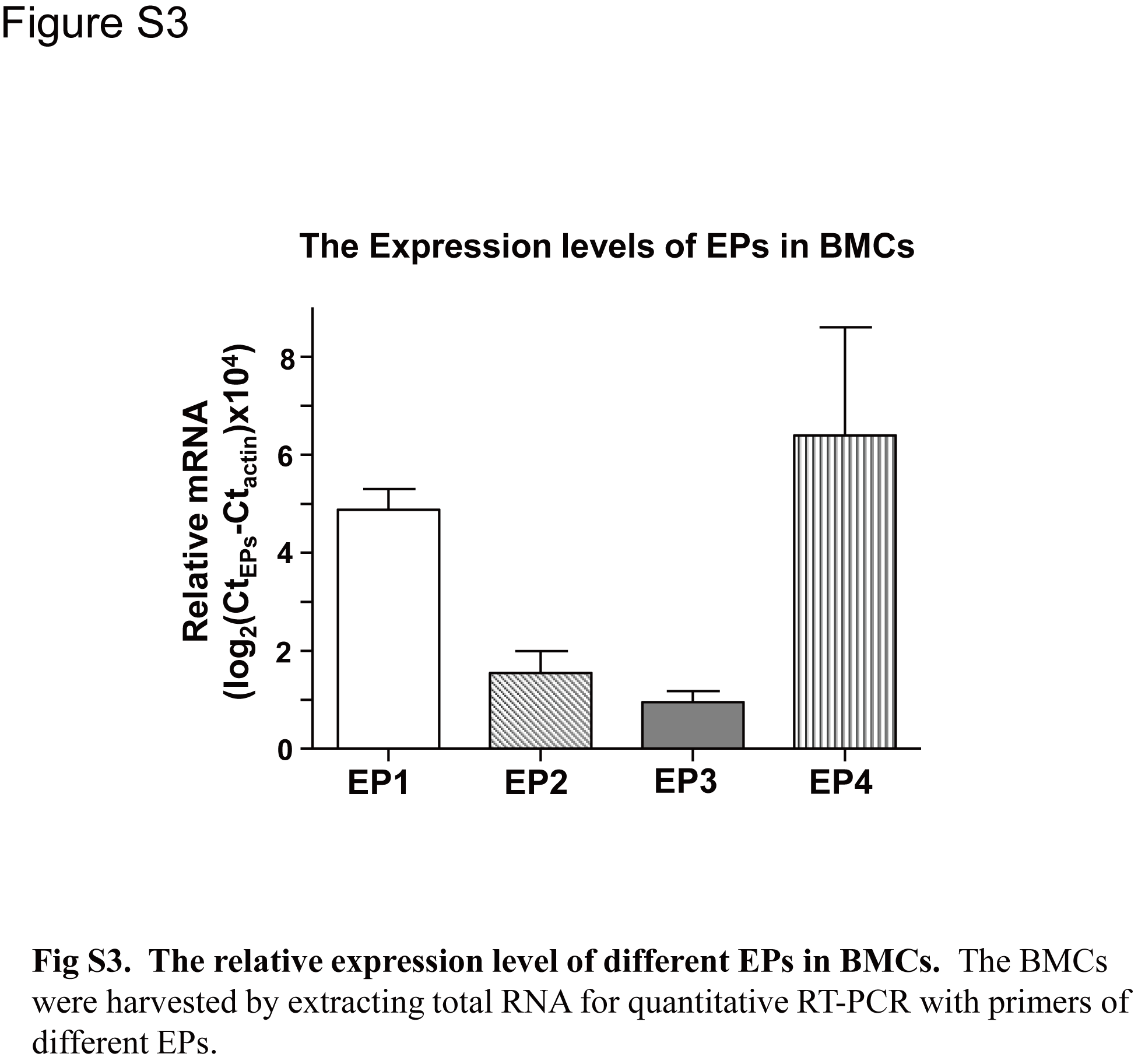

Supplement: Figure S3 — The relative expression level of different EPs in BMCs. The BMCs were harvested by extracting total RNA for quantitative RT-PCR with primers of different EPs. (TIF) [file pone.0023554.s003.tif]

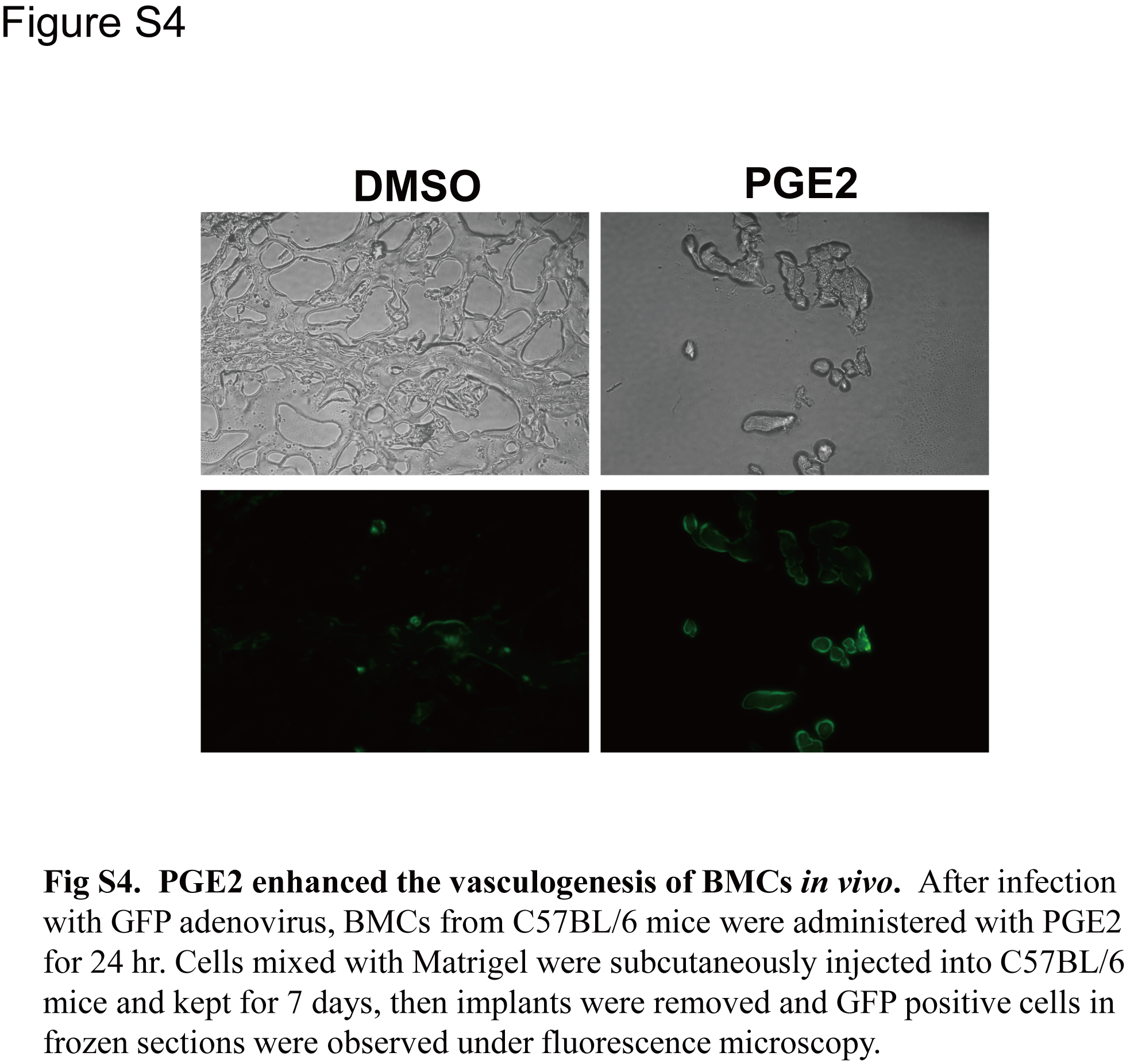

Supplement: Figure S4 — PGE2 enhanced the vasculogenesis of BMCs in vivo. After infection with GFP adenovirus, BMCs from C57BL/6 mice were administered with PGE2 for 24 hr. Cells mixed with Matrigel were subcutaneously injected into C57BL/6 mice and kept for 7 days, then implants were removed and GFP positive cells in frozen sections were observed under fluorescence microscopy. (TIF) [file pone.0023554.s004.tif]
